# Supplementary material for: Vibron-assisted spin excitation in a magnetically anisotropic molecule
Source: Nat Commun. 2020 Apr 1;11:1619. doi: 10.1038/s41467-020-15266-0 (PMC7113279; doi:10.1038/s41467-020-15266-0)
Supplement: Supplementary file 1 — Supplementary Information [file 41467_2020_15266_MOESM1_ESM.pdf]

Supplementary Information to: "Vibron-assisted spin excitation in a magnetically anisotropic molecule"

N. Bachellier,[1] B. Verlhac,[1,*] L. Garnier,[1] J. Zaldívar,[2] C. Rubio-Verdú,[2] P. Abufager,[3] M. Ormaza,[1,4] D.-J. Choi,[5,6] M.-L. Bocquet,[7] J.I. Pascual,[2,6] N. Lorente,[5,8] and L. Limot[1,†]

[1] *Université de Strasbourg, CNRS, IPCMS, UMR 7504, F-67000 Strasbourg, France*
[2] *CIC nanoGUNE, 20018 Donostia-San Sebastián, Spain*
[3] *Instituto de Física de Rosario, Consejo Nacional de Investigaciones Científicas y Técnicas (CONICET) and Universidad Nacional de Rosario, Av. Pellegrini 250 (2000) Rosario, Argentina*
[4] *Universidad del País Vasco, Dpto. Fsica Aplicada I, E-20018 San San Sebastián, Spain*
[5] *Centro de Física de Materiales (CFM), 20018 Donostia-San San Sebastián, Spain*
[6] *Ikerbasque, Basque Foundation for Science, Bilbao, Spain*
[7] *PASTEUR, Département de Chimie, Ecole Normale Supérieure, PSL Research University, Sorbonne Universités, UPMC Univ. Paris 06, CNRS, 75005 Paris, France*
[8] *Donostia International Physics Center (DIPC), 20018 Donostia-San Sebastián, Spain*

(Dated: January 31, 2020)

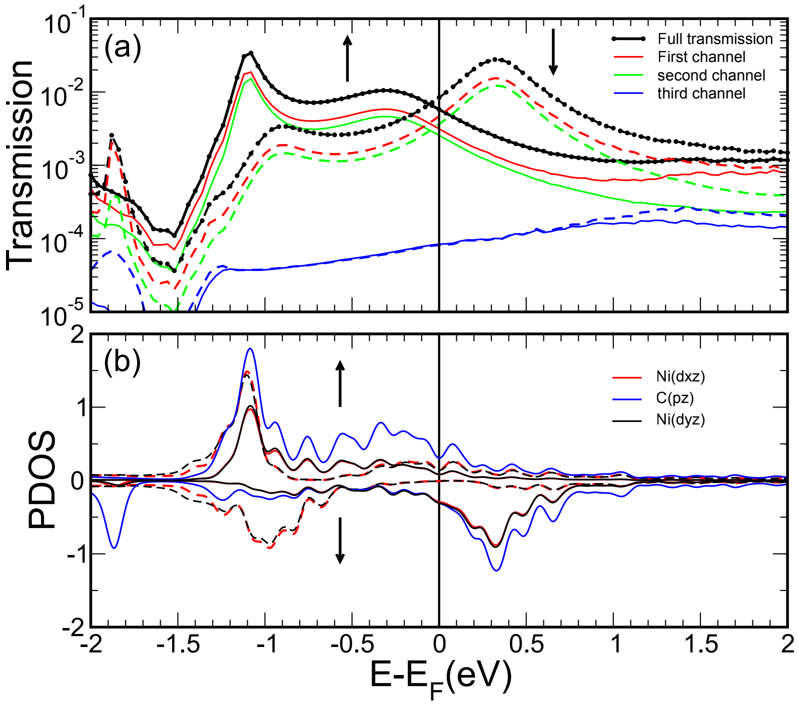

Supplementary Figure 1. **DFT calculations.** (a) Full elastic transmission (black dots) and the transmission of each eigenchannel mainly originating in the π orbitals of the Nc molecule (solid line: spin up, dashed line: spin down). The third eigenchannel (blue lines) negligibly contributes to the total transmission. (b) Projected density of states onto Ni($3d_{xz}$), Ni($3d_{yz}$), C($2p_z$) atomic orbitals (the dashed lines correspond to the Ni-adatom d-states). The frontier π molecular orbitals of Nc have contributions from Ni($3d_{xz}$), Ni($3d_{yz}$) and C($2p_z$) atomic orbitals. The calculations are performed for a tip-molecule distance of 4.9 Å as measured from the tip apex atom to the topmost molecular C. For energies that are accessible with the STM, the transmission is controlled by two eigenchannels that are themselves the contributions of Lorentzian-like functions as can be seen following the transmission curves of the first and second channels. These Lorentzian-like functions energetically match the projected density of states corresponding to the two π molecular orbitals of Nc. For electron energies below -0.8 eV, the transmission involves d-electron channels of the Ni adatom.

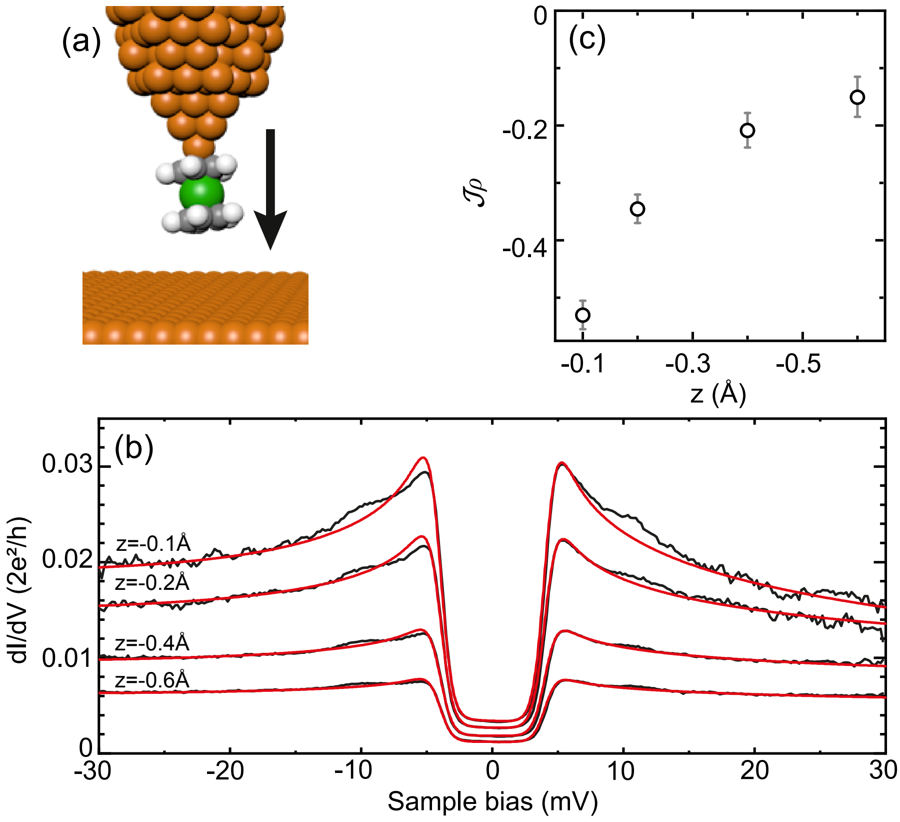

Supplementary Figure 2. **Kondo-like effect of nickelocene.** (a) Sketch of a metal tip functionalized by a Nc molecule. (b) dI/dV spectra at various z (indicated on the figure), along with their simulation based on a dynamical scattering model where we take into account only electron scattering [1] (solid red lines). $z = 0$ corresponds to a nickelocene in contact with the Cu(100) surface. (c) Kondo scattering parameters $\mathcal{J}\rho$ extracted from the simulations in panel (b).

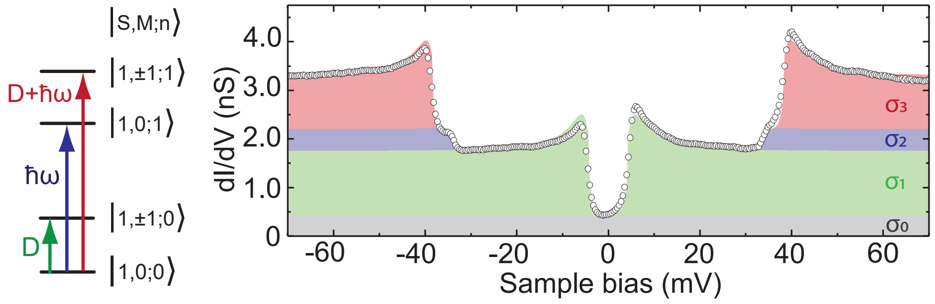

Supplementary Figure 3. **Multi-component fit of the NiNc spectrum.** dI/dV spectrum measured at $T = 2.4$ K (open circles) and multi-component fit based on the phenomenological model described in the main text. The colored areas show contributions to the fit due to elastic tunneling (noted **0**), to spin-dependent inelastic tunneling (**1** and **3**) and to vibrational inelastic tunneling (**2**). Feedback loop opened at -80 mV and 200 pA.

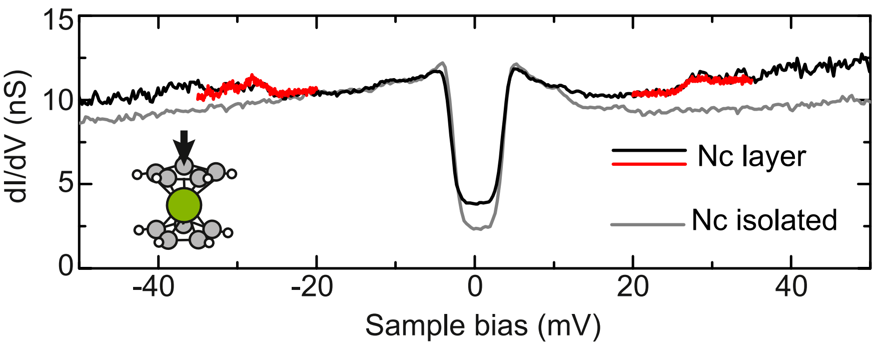

Supplementary Figure 4. **Local spectra of isolated and layer-integrated Nc.** dI/dV spectrum acquired above the center of a layer-integrated Nc (solid black line) and of an isolated Nc (solid gray line). An inelastic step is evidenced at ± 28 mV. The solid red line corresponds to high-resolution data acquired between -35 mV and -20 mV, and between 20 mV and 35 mV. The feedback loop was opened at -30 mV and 300 pA for all spectra. Inset: Arrow indicates the position where the spectra were acquired.

Tunnel current (pA)	ϵ_1 (meV)	ϵ_2 (meV)	ϵ_3 (meV)
50	4.6	33.4	37.6
500	4.7	32.6	37.1
1000	4.7	31.9	36.8

Supplementary Table 1. **Inelastic tunneling in NiNc versus tip-molecule distance.** Energy onsets extracted from the dI/dV spectra of NiNc that are shown in Fig. 2c. The position of peak (dip) **1** is nearly constant with tunnel current, *i.e.* tip-molecule distance. If present, changes in the magnetic anisotropy of NiNc due to the electric field are therefore below detectability [2, 3].

ϵ_1 (meV)	ϵ_2 (meV)	ϵ_3 (meV)	$\epsilon_1 + \epsilon_2$ (meV)
4.6	32.8	37.1	37.4
4.2	34.5	39.0	38.7
4.3	33.9	38.0	38.2
4.9	34.0	38.0	38.9
3.7	34.0	37.7	37.7
4.1	34.1	38.6	38.2

Supplementary Table 2. **Energy onsets for inelastic tunneling in NiNc.** Energy onsets extracted from the dI/dV spectrum of six NiNc complexes using the fit shown in Supplementary Fig. 3.

σ_1/σ_0	σ_2/σ_0	σ_3/σ_0	$(\sigma_1\sigma_2)/\sigma_0^2$
2.64	0.89	2.28	2.36
2.70	0.75	1.83	2.04
2.80	1.33	3.49	3.73
1.91	0.86	1.41	1.64
3.33	0.78	2.00	2.59
2.01	0.90	1.54	1.83

Supplementary Table 3. **Inelastic conductances of NiNc.** Step amplitudes extracted from the dI/dV spectrum of six NiNc complexes using the fit shown in Supplementary Fig. 3.

Supplementary Note 1. Kondo scattering of nickelocene

In a previous study, we functionalized the copper apex of a tip with a single nickelocene molecule [Nc-tip here after, Supplementary Fig. 2(a)] [4]. We showed that the spin excitation spectrum of nickelocene is nearly identical to that of nickelocene adsorbed on a Cu(100) surface. Supplementary Fig. 2(b) presents a set of dI/dV spectra acquired with a Nc-tip positioned at various distances z from the Cu(100) surface —the distances are determined through current-versus-z curves (not shown). The distance $z = 0$ corresponds to a nickelocene in contact with the Cu(100) surface. The inelastic thresholds remain constant with z, indicating that the magnetic anisotropy D is constant with the distance. An enhancement of the differential conductance at voltages corresponding to the excitation threshold is however observed, which is increasingly pronounced as the contact point is approached ($z > -0.5$ Å). This cusp is attributed to electron scattering involving Kondo-like phenomena [5, 6].

To model the Kondo effect we use the dynamical scattering model of Ref. 1 and estimate the dimensionless coupling constant $\mathcal{J}\rho$ describing the strength of the Kondo exchange interaction, $\mathcal{J}$, between the localized spin and the electron density ρ of the substrate near the Fermi level [7]. The simulations are presented as red lines in Supplementary Fig. 2(b), while Supplementary Fig. 2(c) presents the values of $\mathcal{J}\rho$. The agreement with the experimental spectra is highly satisfactory demonstrating that the cusp is governed by the hybridization of nickelocene with the surface. In particular, the value of $\mathcal{J}\rho$ found for NiNc in the main text agrees with the values reported in Supplementary Fig. 2(c) for nickelocene.

Supplementary Note 2. Multi-component fit

The spin Hamiltonian [Eq. (2)] in the main text qualitatively explains all the features in the experimental dI/dV spectrum. However, for a quantitative estimate of the step amplitudes, we use an extended phenomenological model. The first spin excitation of amplitude σ_1 can be accurately reproduced with a fit based on the dynamical scattering model of Ref. 1 [solid red line in Fig. 2(a) of the main text]. The fit yields an axial magnetic anisotropy of $D = 4.6 \pm 0.2$ meV, a coupling of $\mathcal{J}\rho = -0.4 \pm 0.2$ between the localized Nc spin and the substrate electrons, and a spin-conserving potential scattering of $\mathcal{U} = -0.1 \pm 0.1$; the latter accounts for the weak asymmetry of the line shape.

To fit the full spectrum (Supplementary Fig. 3), we use a constant elastic contribution σ_0, a vibrational step of amplitude σ_2 accounted for following Ref. 8. The two spin excitations of amplitudes σ_1 and σ_3 are accounted for using again Ref. 1; we use the same fitting parameters for the two spin excitations, except for their amplitudes σ_1 and σ_3. For simplicity, we set $\mathcal{U} = 0$ thereby neglecting the weak asymmetry of the line shape, without loss of generality. The fit to the data is presented in Supplementary Fig. 3 and is highly satisfactory. The energy onsets of these steps (noted ϵ_1, ϵ_2 and ϵ_3) are related by $|\epsilon_3| = |\epsilon_1| + |\epsilon_2|$ as shown in Supplementary Tab. 2. The amplitudes show some dependency on the NiNc complexes investigated, but their relative ratios obey $\sigma_3/\sigma_0 \approx (\sigma_2/\sigma_0)(\sigma_1/\sigma_0)$ (Supplementary Tab. 3).

Supplementary references

* verlhac@ipcms.unistra.fr
† limot@ipcms.unistra.fr

[1] M. Ternes, New J. Phys. **17**, 063016 (2015).
[2] J. Hu and R. Wu, Phys. Rev. Lett. **110**, 097202 (2013).
[3] Y. Zhang, J. Chem. Phys. **146**, 194705 (2017).
[4] M. Ormaza, P. Abufager, B. Verlhac, N. Bachellier, M. L. Bocquet, N. Lorente, and L. Limot, Nat. Commun. **8**, 1974 (2017).
[5] A. Hurley, N. Baadji, and S. Sanvito, Phys. Rev. B **84**, 115435 (2011).
[6] R. Korytár, N. Lorente, and J.-P. Gauyacq, Phys. Rev. B **85**, 125434 (2012).
[7] P. Jacobson, T. Herden, M. Muenks, G. Laskin, O. Brovko, V. Stepanyuk, M. Ternes, and K. Kern, Nat. Commun. **6**, 8536 (2015).
[8] J. Lambe and R. C. Jaklevic, Phys. Rev. **165**, 821 (1968).
